# Supplementary figures and images for: Growing Glycans in Rosetta: Accurate de novo glycan modeling, density fitting, and rational sequon design
Source: PLoS Comput Biol. 2024 Jun 24;20(6):e1011895. doi: 10.1371/journal.pcbi.1011895 (PMC11288642; doi:10.1371/journal.pcbi.1011895)

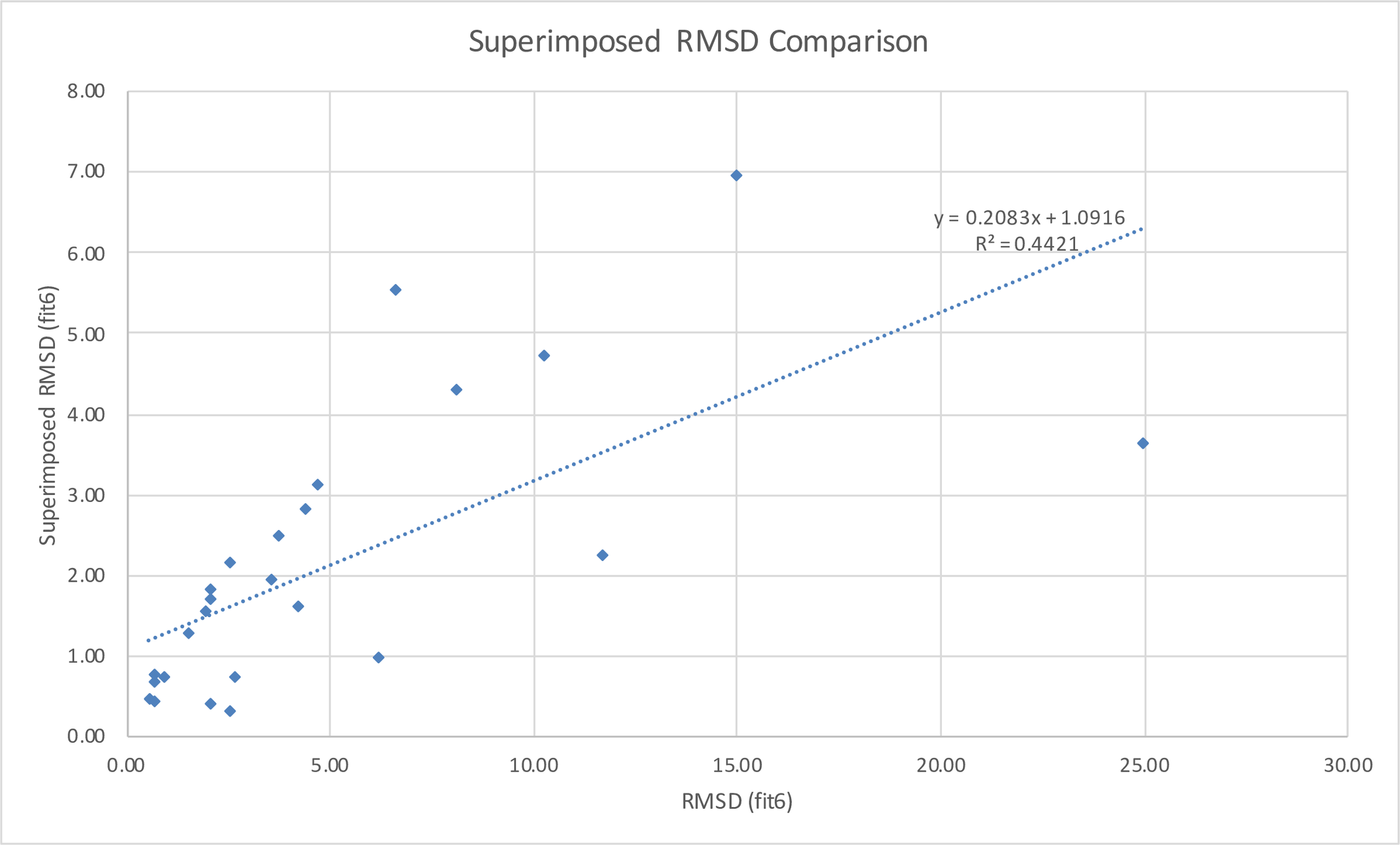

Supplement: S1 Fig — (TIF) [file pcbi.1011895.s010.tif]

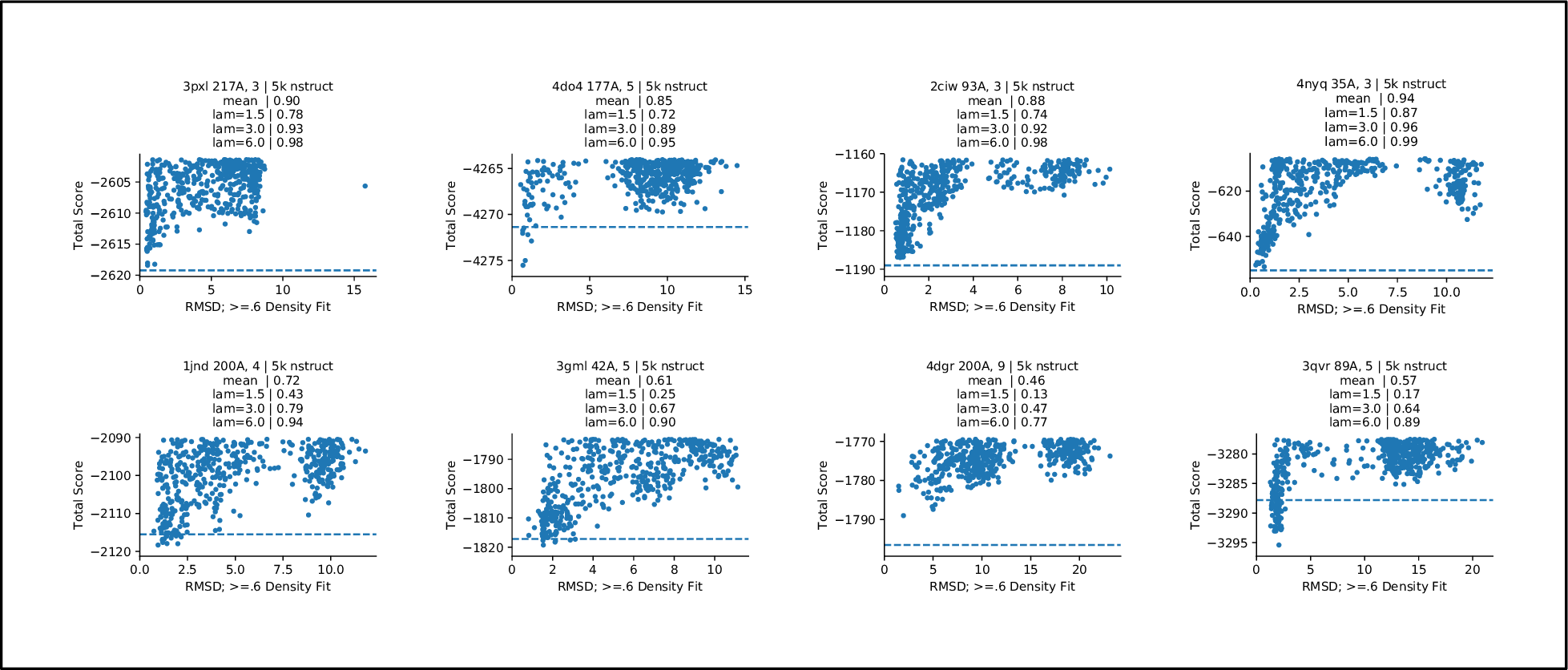

Supplement: S2 Fig — Shown is the top 10% of models by total energy. Blue line is the scored native structure with symmetry. (TIF) [file pcbi.1011895.s011.tif]

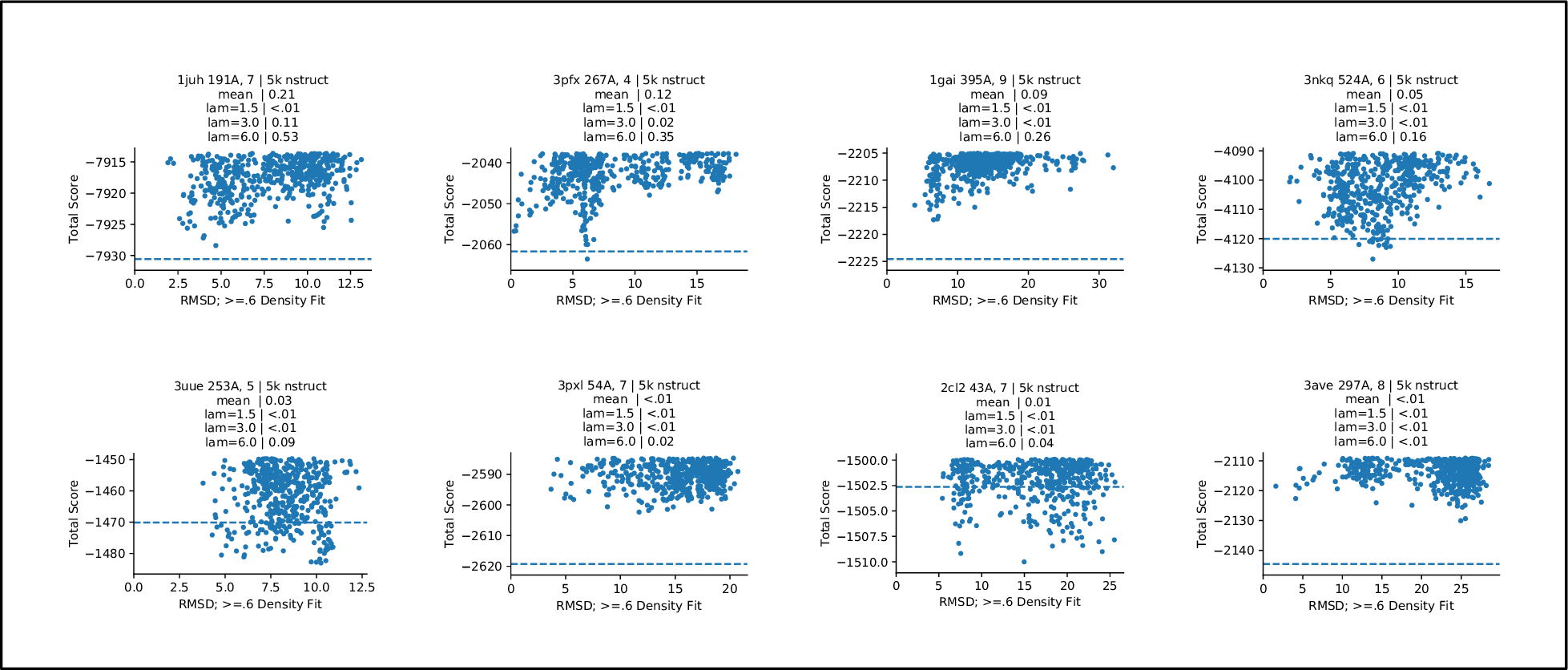

Supplement: S3 Fig — Shown is the top 10% of models by total energy. Blue line is the scored native structure with symmetry. (TIF) [file pcbi.1011895.s012.tif]

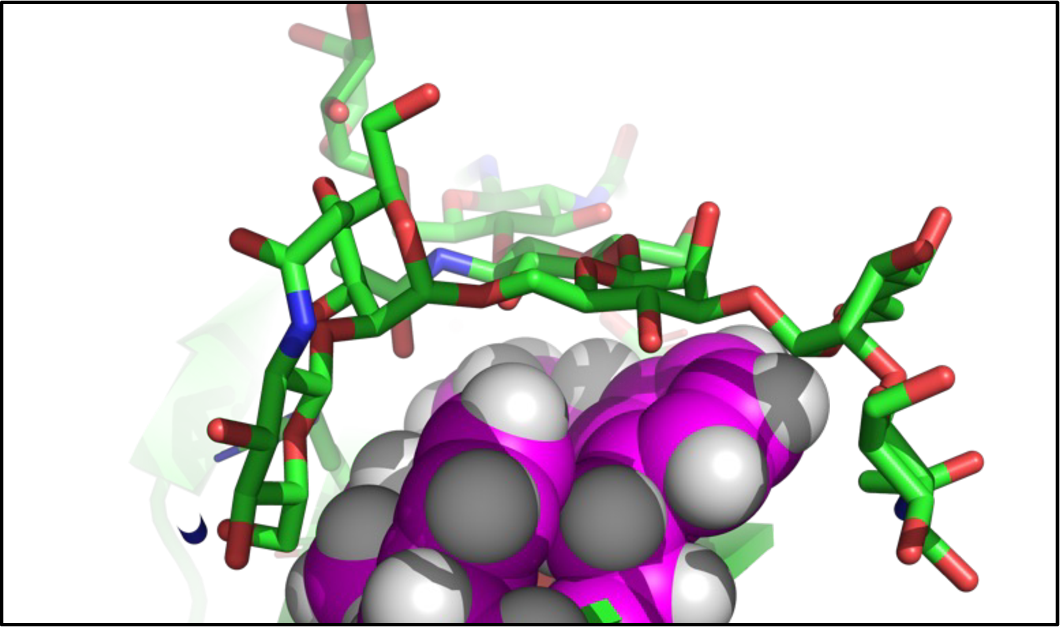

Supplement: S4 Fig — F241, 243F, 262V, and 264V are shown as spheres at the glycan interface. (TIF) [file pcbi.1011895.s013.tif]

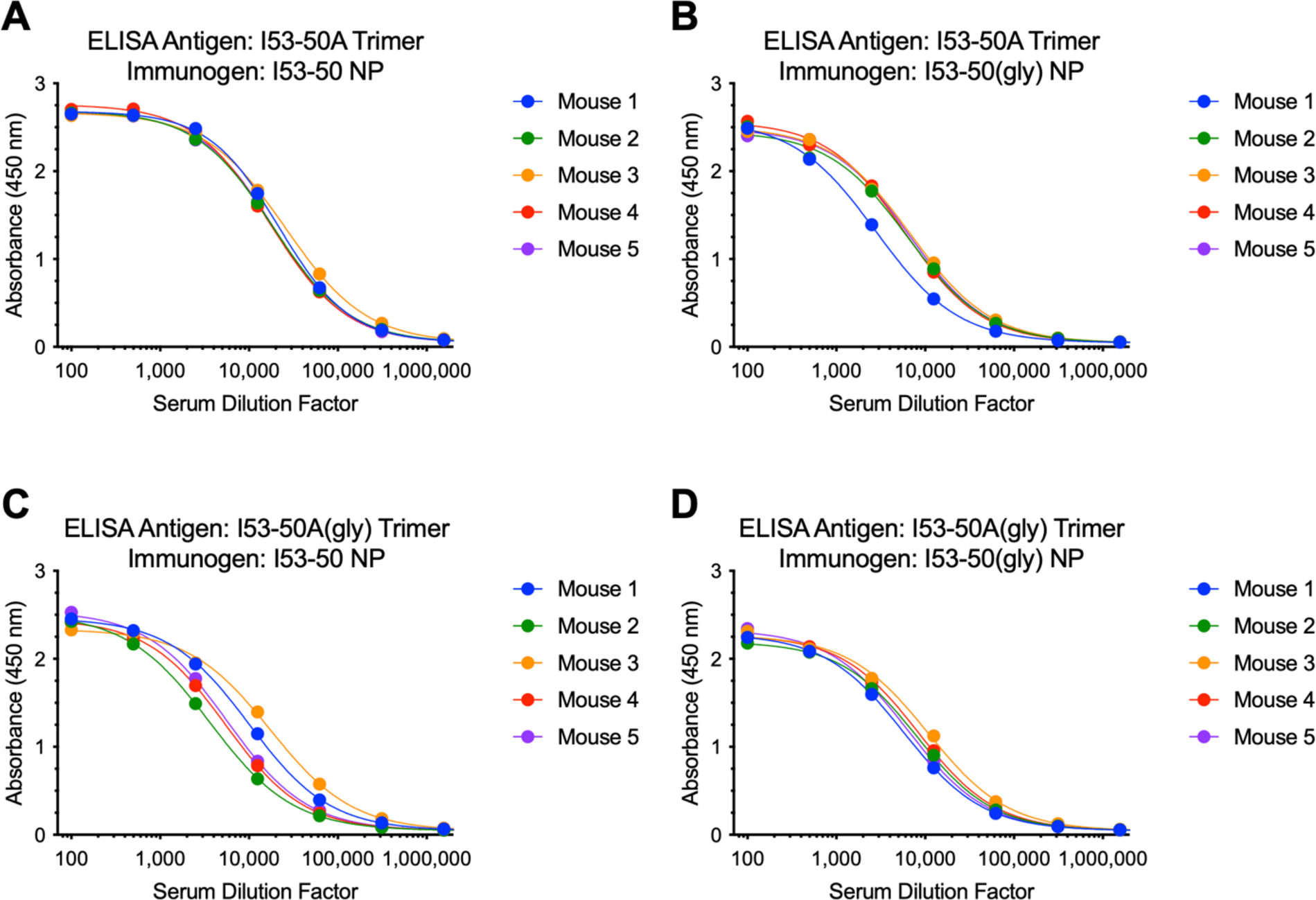

Supplement: S5 Fig — (a,b) anti-I53-50A trimer or (c,d) anti-I53-50(gly) trimer antibody responses from mice immunized with (a,c) I53-50 nanoparticles (NP) or (b,d) I53-50(gly) NP. (TIF) [file pcbi.1011895.s014.tif]

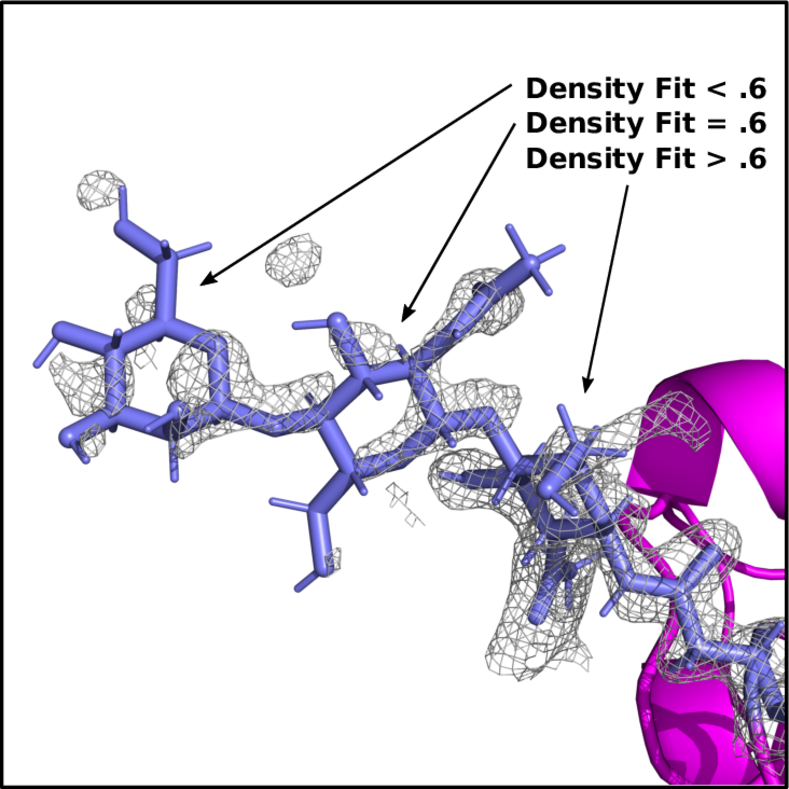

Supplement: S6 Fig — (TIF) [file pcbi.1011895.s015.tif]

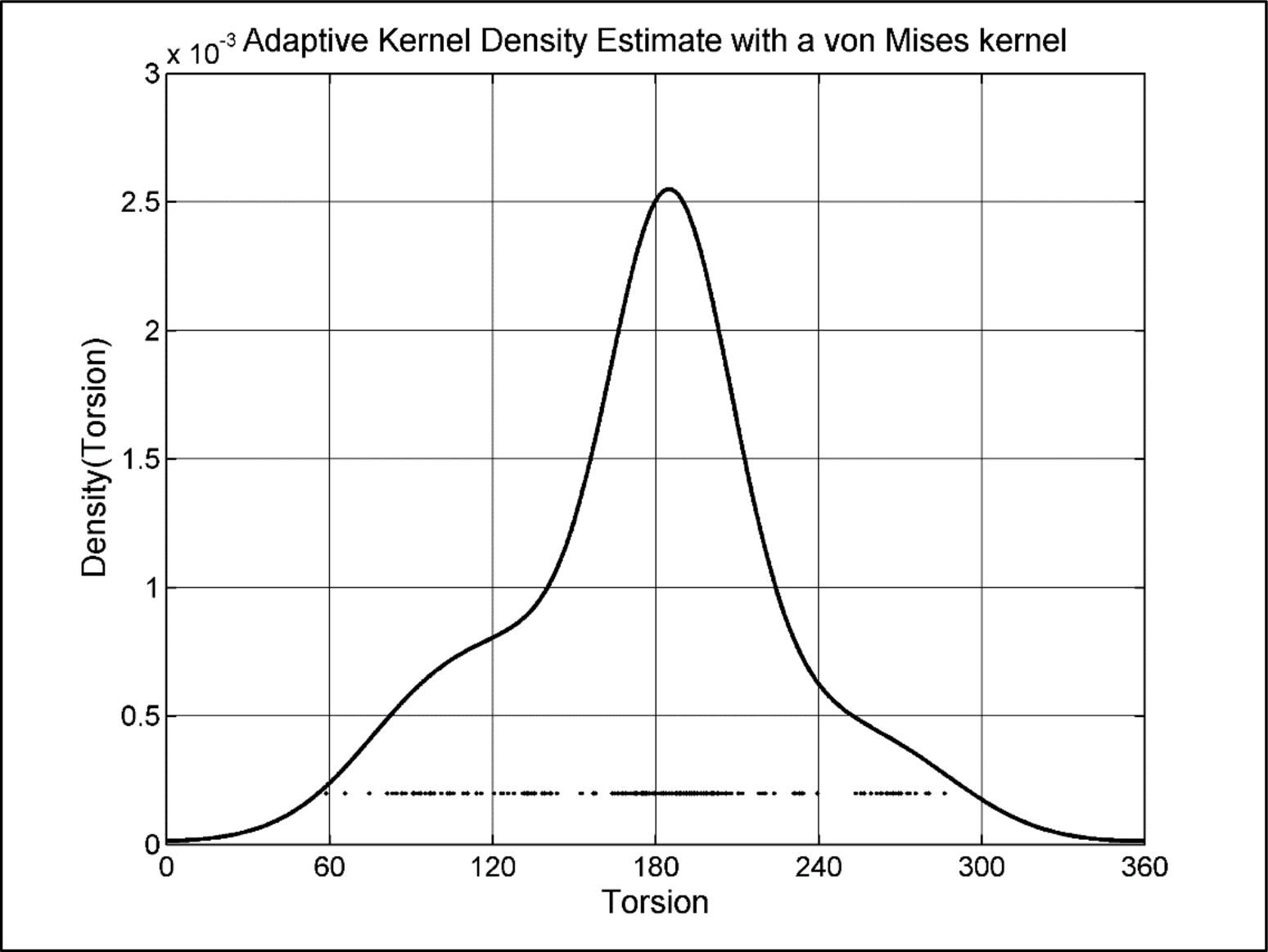

Supplement: S7 Fig — The von Mises kernel allows for continuous circular description of the torsion angle distribution. The experimental angles from a sample are shown with small dots at the bottom. Such 1-D density estimates were performed for each torsion comprising a glycan-glycan or amino-acid-glycan linkage type. These 64 linkage types can be found in the resulting conformer table included in S1 Data. (TIF) [file pcbi.1011895.s016.tif]

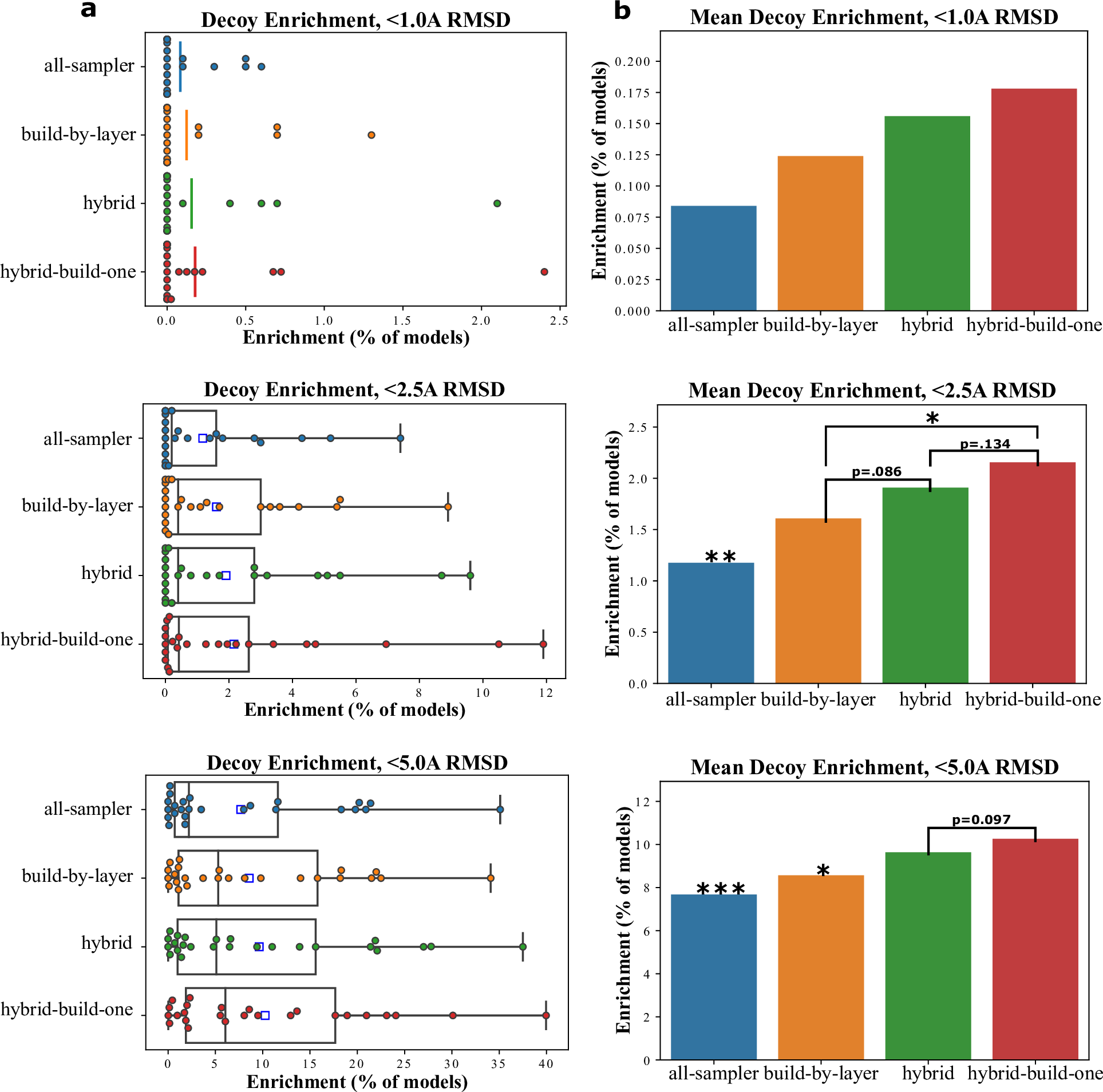

Supplement: S8 Fig — All experiments were conducted with the same total amount of sampling. a. Boxplots at decoy enrichments of <1A, <2.5A, and <5.0A. First figure has mean only since most are grouped at zero. b. decoy enrichments of <1A, <2.5A, and <5.0A. Asterisks indicate statistically significant differences through paired t-test. Asterisk above bar indicate statistical significance with all other groups. *,p < .05; **,p < .005; ***,p < .0005. (TIF) [file pcbi.1011895.s017.tif]

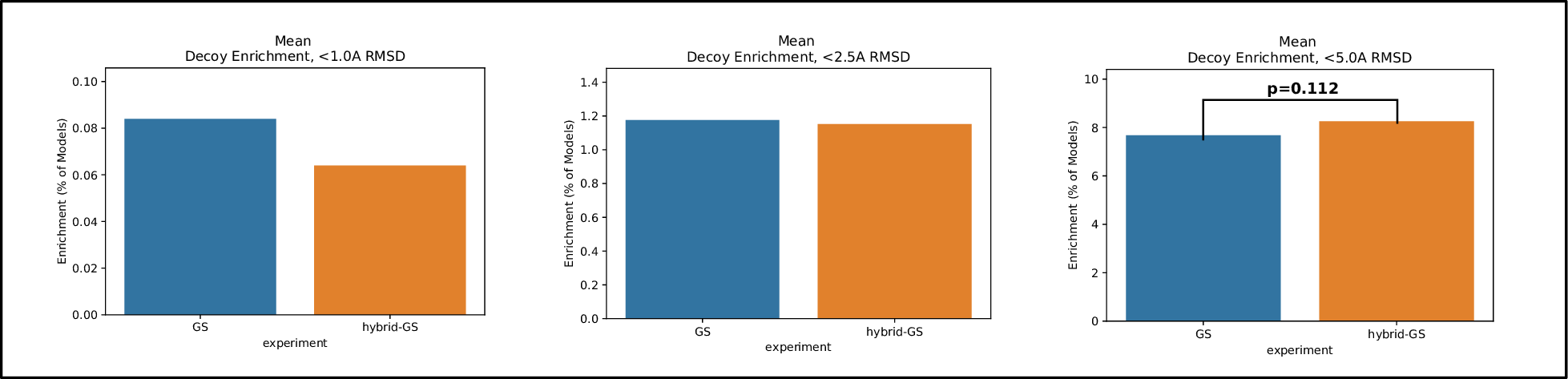

Supplement: S9 Fig — (TIF) [file pcbi.1011895.s018.tif]

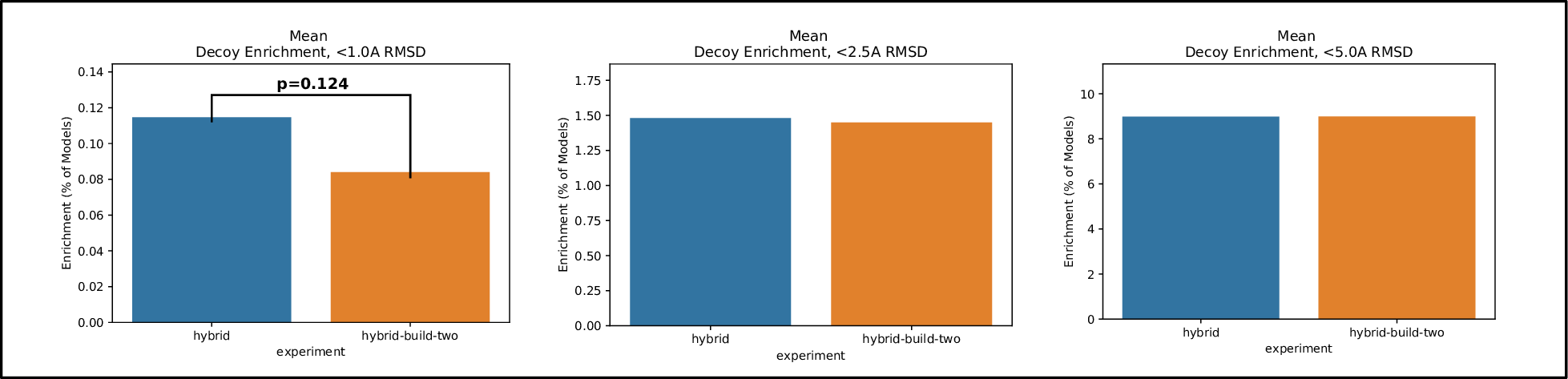

Supplement: S10 Fig — (TIF) [file pcbi.1011895.s019.tif]

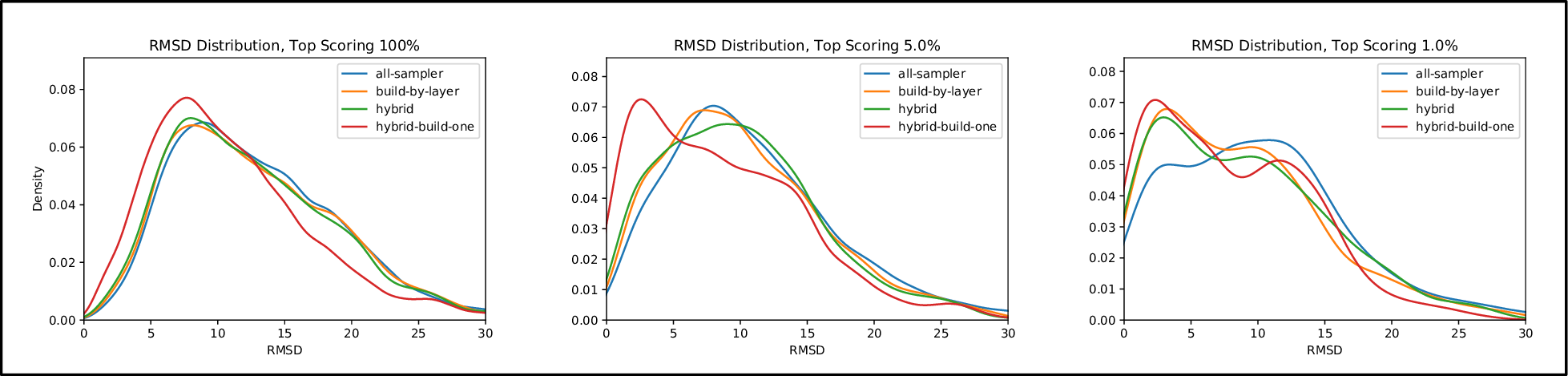

Supplement: S11 Fig — N = 150,000; 37,500 per experiment. (TIF) [file pcbi.1011895.s020.tif]

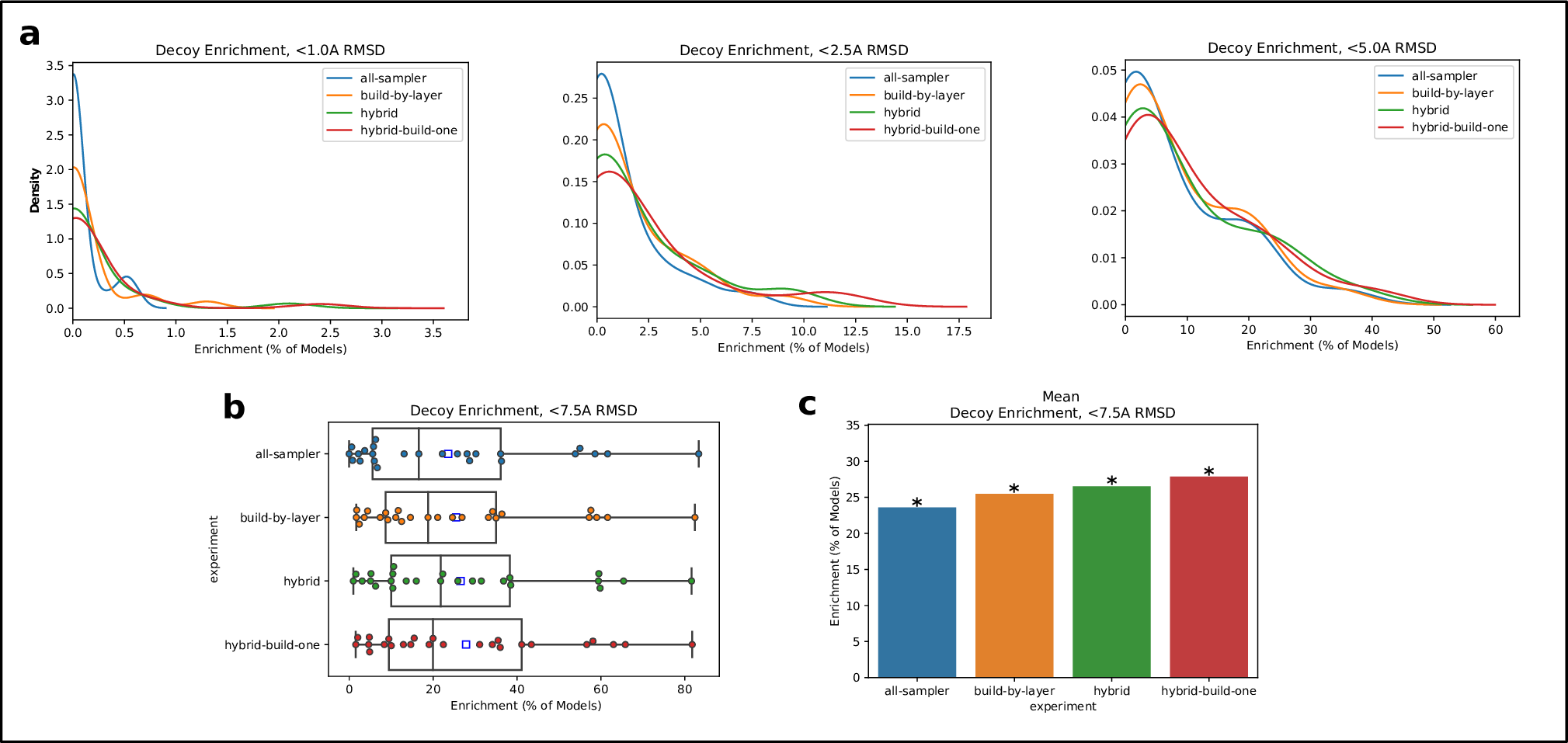

Supplement: S12 Fig — a. Kernel Density Estimates of enrichment per input model for each major kinematic experiment. b. Box plot of enrichment of each input model per experiment less than 7.5 A RMSD to the native crystal structure. C. Means of B, with paired t-test, All vs. All. * indicates p < .05. p-value for hybrid-build-one vs. build-by-layer p< .005, while vs. all-sampler p < .0005. (TIF) [file pcbi.1011895.s021.tif]

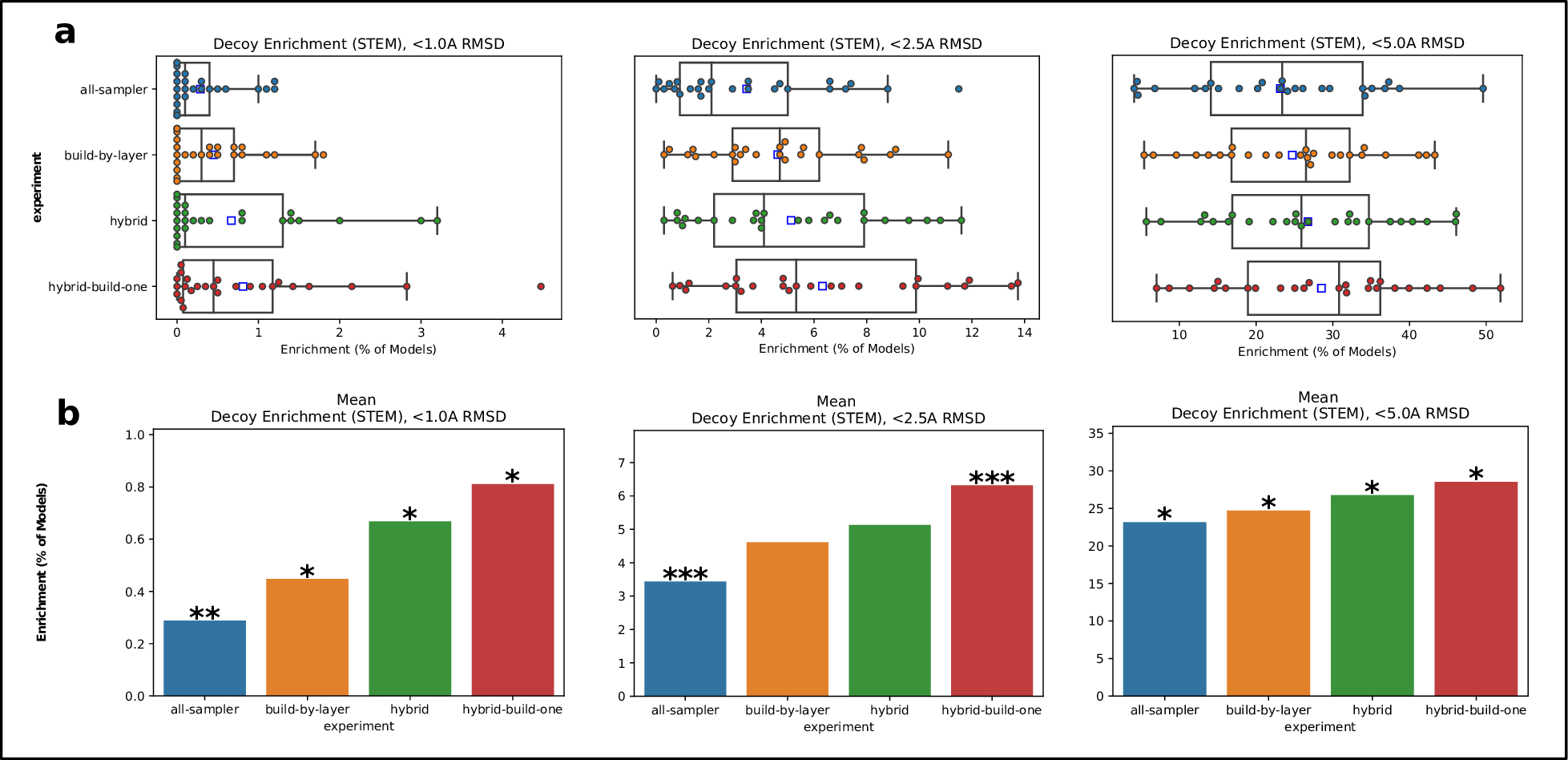

Supplement: S13 Fig — a. Boxplots of each input glycan benchmark at <1.0A, <2.5A, and < 5.0A of the glycan STEM b. Means of panel a. Asterisk above bar indicate statistical significance with all other groups through paired t-test. *|p < .05; **|p < .005; ***|p < .0005. For b <1A, pvalue of all-sampler vs. hybrid-build-one is **. (TIF) [file pcbi.1011895.s022.tif]

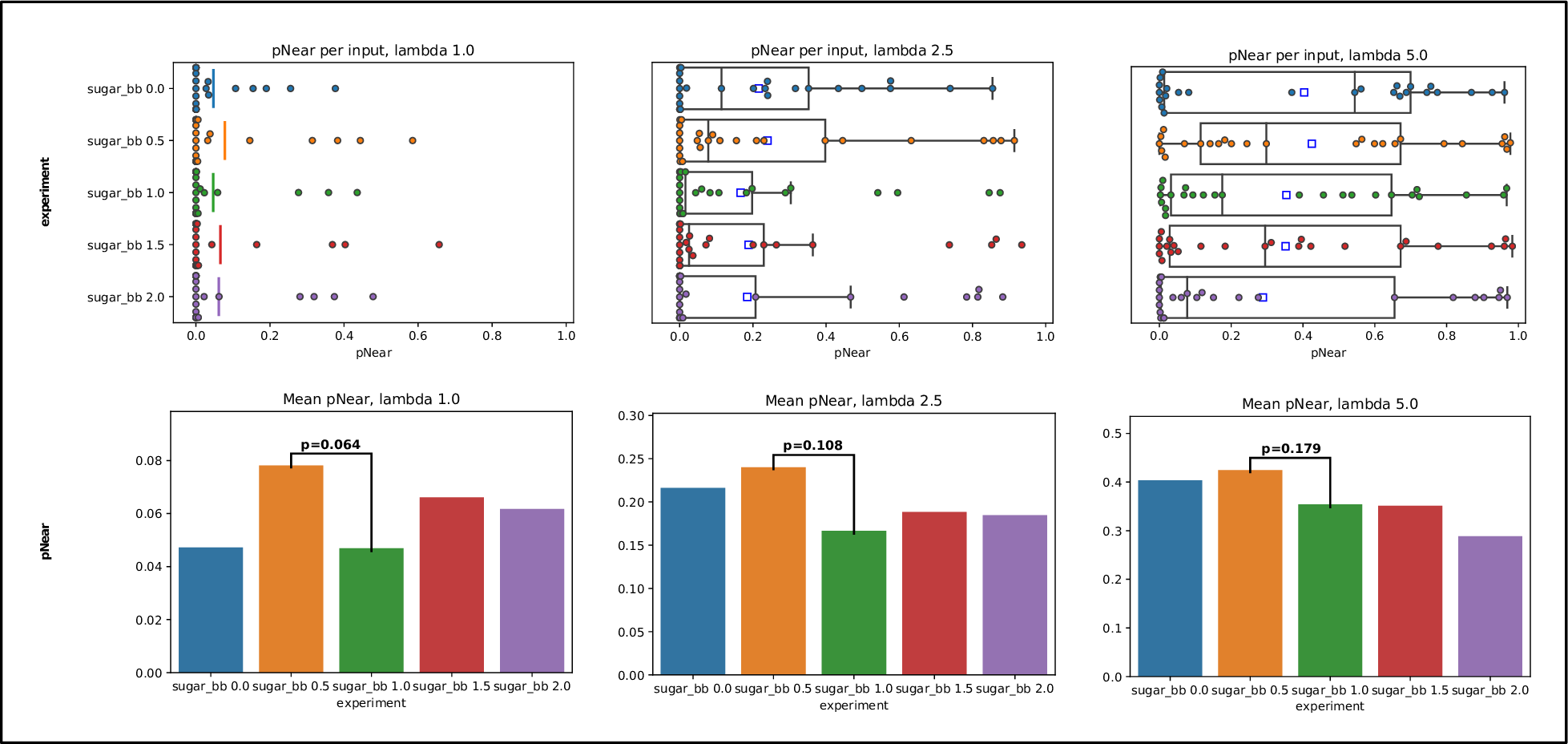

Supplement: S14 Fig — 1000 decoys were produced for each glycan and each experiment for a total of 125k decoys. Note that this is a third less than all other optimization experiments. Boxplots and barplots of PNear metric at each significant lambda are shown. Blue squares indicate mean. Line in box indicates median. Upper left figure shows only means as most datapoints are grouped at 0 and the box could not be seen. Paired T-test results between sugar_bb weight of 1.0 and .5 are indicated. (TIF) [file pcbi.1011895.s023.tif]

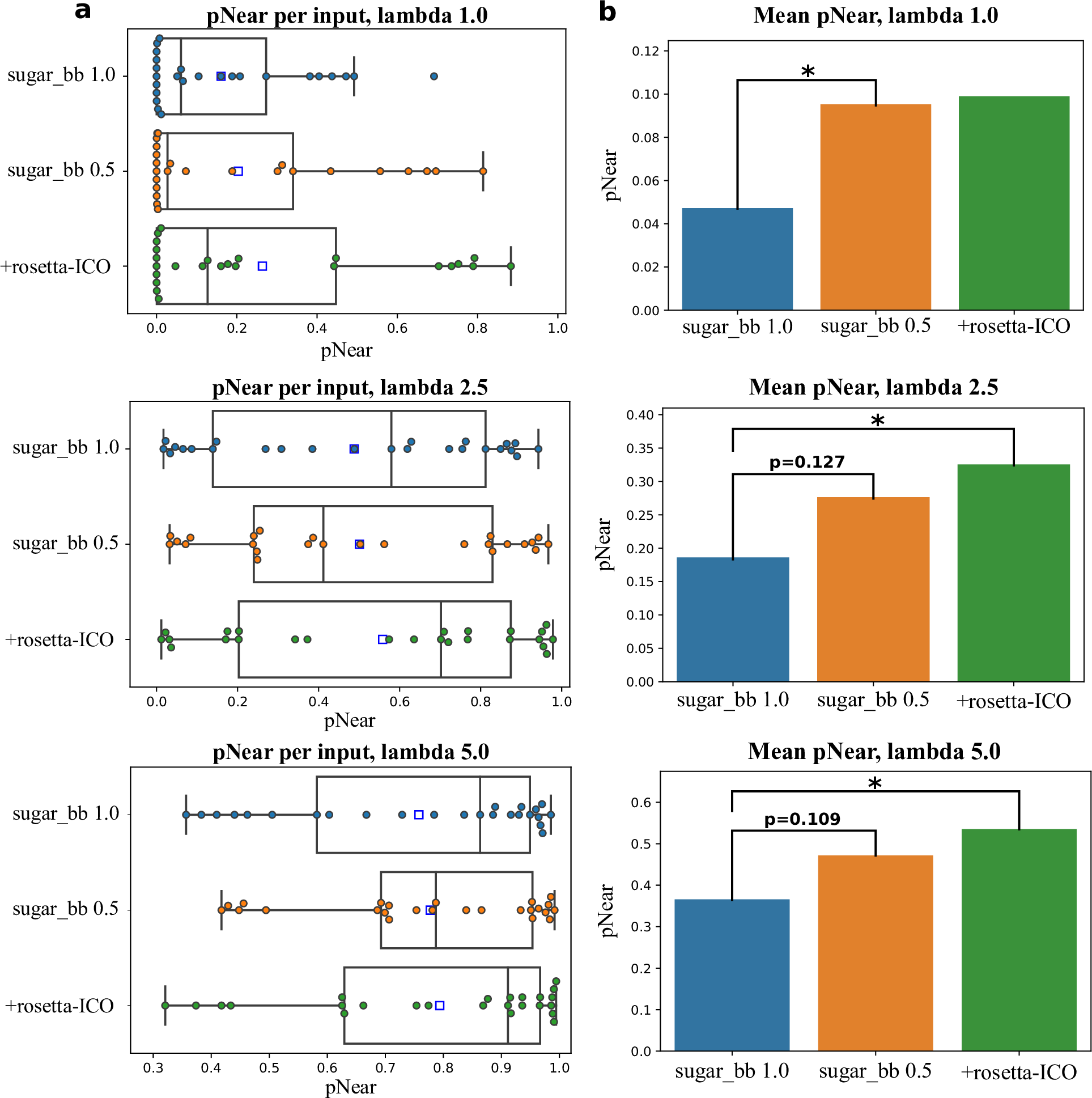

Supplement: S15 Fig — a. Boxplot of pNear values for each benchmark glycan, indicating funnel plot quality for lambdas of 1.0, 2.5, and 5.0 RMSD to native. Higher pNear indicates better near-native discrimination from other decoys. Blue squares indicate mean. b. Means of pNear over each experiment. Significance from paired t-test; * indicates p < .05. (TIF) [file pcbi.1011895.s024.tif]

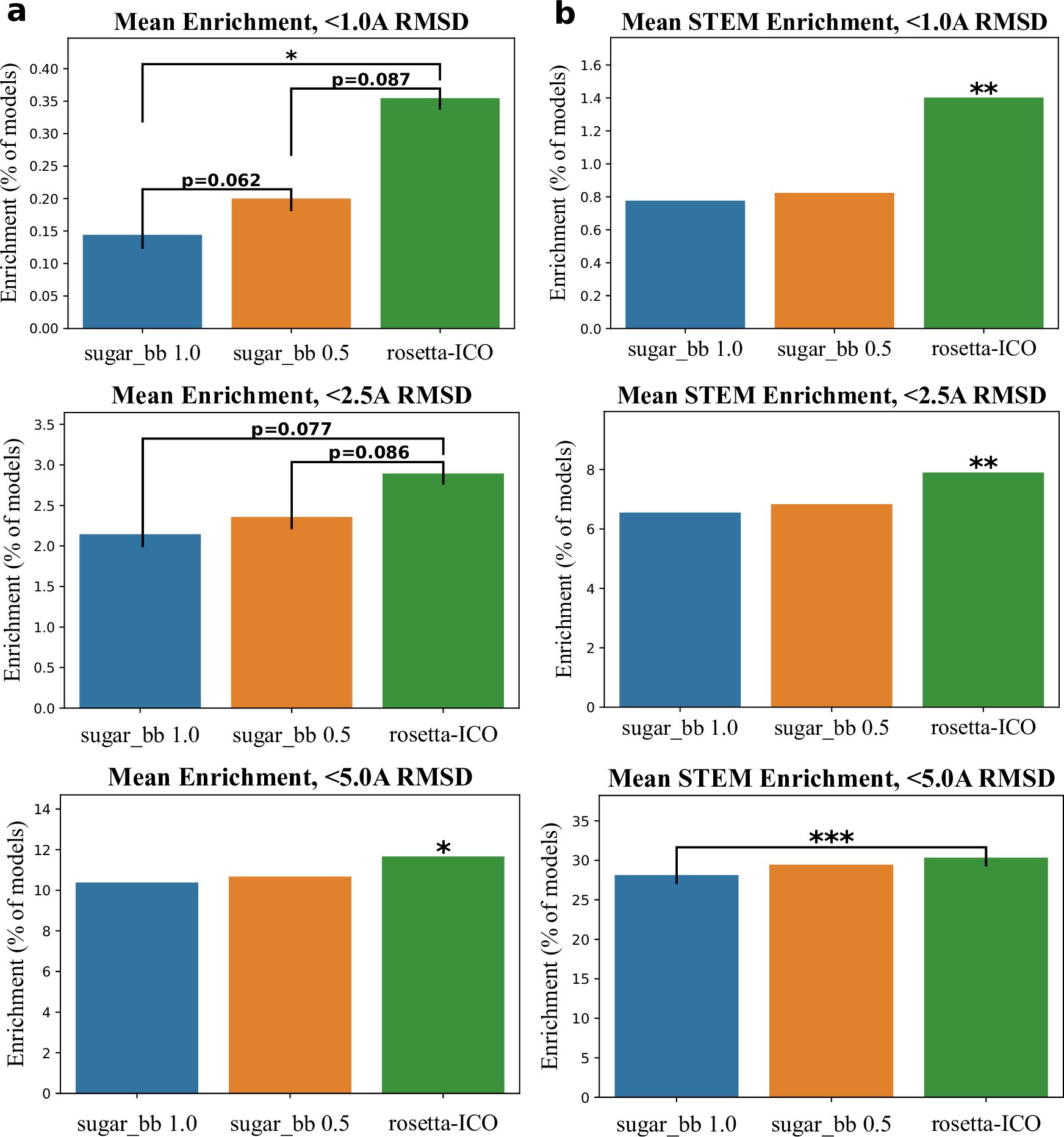

Supplement: S16 Fig — Asterisk above bar indicate statistical significance with all other groups through paired t-test. *|p < .05 **|p < .005 ***|p < .0005 a. Decoy Enrichment in output models at <1.0A, <2.5A, and <5.0A RMSD. b. Decoy Enrichment in output models of the base (STEM) region indicating layers 0 and 1. (TIF) [file pcbi.1011895.s025.tif]

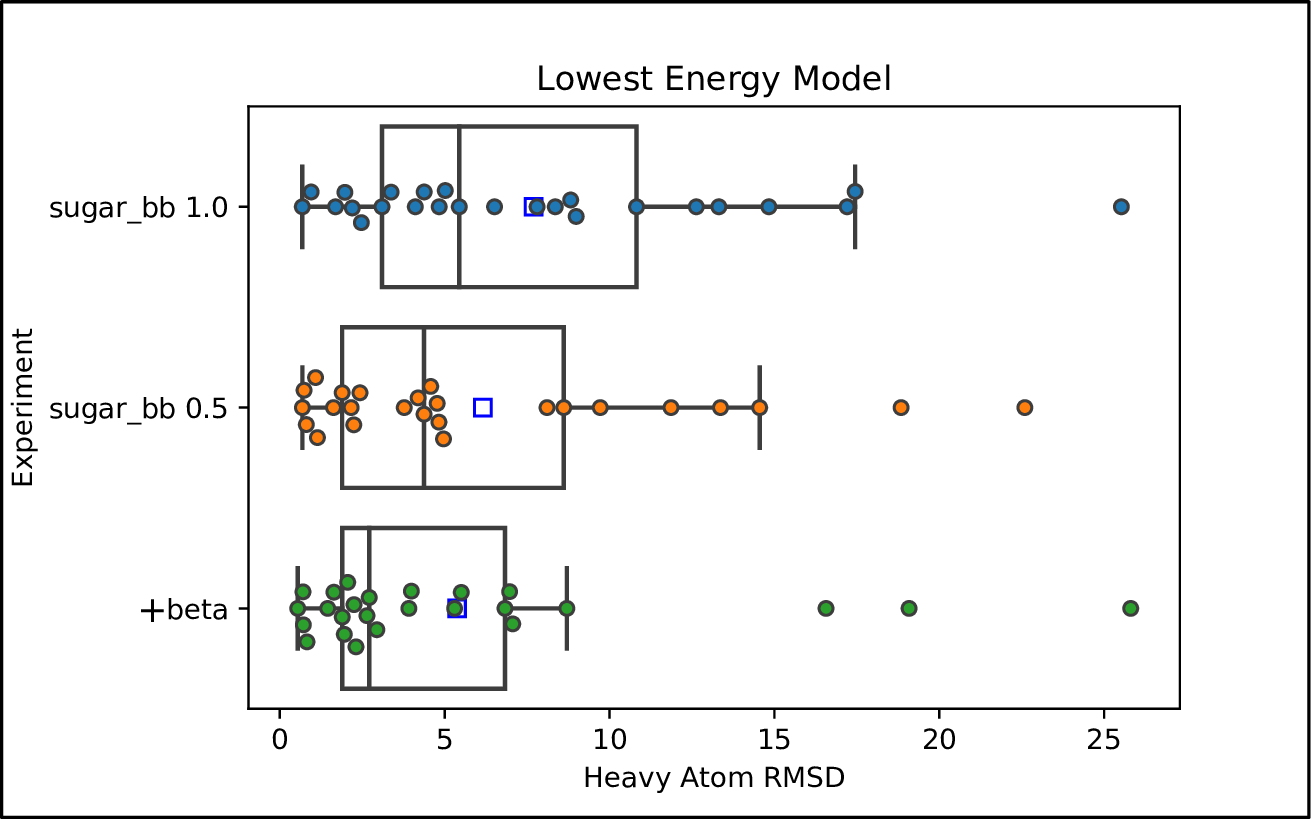

Supplement: S17 Fig — (TIF) [file pcbi.1011895.s026.tif]
